# Women’s Knowledge, Attitudes and Behavior about Maternal Risk Factors in Pregnancy

**DOI:** 10.1371/journal.pone.0145873

**Published:** 2015-12-29

**Authors:** Giuseppe Esposito, Rossella Ambrosio, Francesco Napolitano, Gabriella Di Giuseppe

**Affiliations:** Department of Experimental Medicine, Second University of Naples, Naples, Italy; Nathan Kline Institute and New York University School of Medicine, UNITED STATES

## Abstract

**Background:**

The aims of this study were to assess the levels of knowledge, attitudes and behaviors of women about the main maternal risk factors in pregnancy and to identify the factors linked to the main outcomes of interest.

**Materials and Methods:**

A cross-sectional survey was conducted in 513 pregnant women randomly selected from the gynecological ambulatory services of five hospitals located in Naples, Italy.

**Results:**

Only 42% of women correctly knew all the main maternal risk factors in pregnancy (alcohol, smoking, passive smoking and obesity). Only 21.7% of women were very worried about causing harm to the fetus or child with their risk behaviors, and 22.3% of women reported smoking during pregnancy. Approximately one-third of women (28.9%) reported regularly drinking alcohol before pregnancy and 74.8% of these women reported stopping drinking alcohol during pregnancy. However, only 27.3% of women who were drinking alcohol during pregnancy had the intention of stopping. Only 43.7% of women indicated that during ambulatory gynecological examinations they received information from physicians about the possible damage resulting from all the main risk factors in pregnancy (alcohol, smoking, passive smoking and obesity).

**Conclusion:**

The results indicate that pregnant women lack knowledge regarding the main maternal risk factors. Pregnant women claim to receive little information during gynecological examinations and, therefore, some continue to smoke and drink alcohol during pregnancy. Our results suggest an urgent need for the design of interventions to improve women’s levels of knowledge and to promote appropriate behavior in relation to the major risk factors in pregnancy.

## Introduction

The preconception period is considered an important time for women’s health and an opportunity to develop a healthy lifestyle that can be useful both for the health of the mother and the newborn baby [1,2]. Although it has been well documented that there is a need to implement interventions to promote appropriate behaviours in women of reproductive age before the conception and that maternal risk factors should be identified and modified also before conception [3–6], the Healthy People 2020 strategy still indicated that pregnancy is a good time to identify existing maternal risk factors. It also states that increased knowledge among women about maternal risk factors may result in immediate benefits by reducing adverse events in pregnancy and birth and long-term benefits for the health of mothers and children [7].

Indeed, it is well established that maternal behaviors and several conditions are associated with adverse pregnancy outcomes. In particular, lifestyle factors such as tobacco and alcohol use in pregnancy increase the risk of low birth weight, preterm delivery and perinatal mortality [8–13]. Moreover, maternal wieght problems, obesity, gestational diabetes and failure to take folic acid supplementation were associated with an increased risk of pre-eclampsia, neurological, cardiac and orofacial defects, high birth weight and stillbirth [14–20].

Several studies have assessed the knowledge and behaviors of reproductive aged women and pregnant women with regard to some individual risk factors in pregnancy such as smoking [21–23], alcohol consumption [24,25], oral health [26] and obesity [27,28]. However, very few studies have examined several risk factors simultaneously [29–31]. Therefore, the present survey, conducted with a representative sample of pregnant women, had two primary aims. The first aim was to assess the level of knowledge, attitudes and behaviors of women about the main maternal risk factors in pregnancy. The second aim was to identify the factors linked to the knowledge and attitudes of women towards the main maternal risk factors in pregnancy and to profile women who smoke during pregnancy.

## Materials and Methods

From October 2012 to March 2013, a cross-sectional survey was conducted in a sample of 513 pregnant women of all ages and in any week of pregnancy. They were randomly selected from the gynecological ambulatory services of five hospitals located in Naples, Italy. The sample was established from stratified cluster sampling in a single stage. Before starting the survey, the hospital director and the head of the department of gynecology of each hospital received a letter with the description of the study and approval was obtained.

The survey was carried out through a self-administered anonymous questionnaire. Pregnant women had received the questionnaire in the waiting room of ambulatory services and twenty minutes was given to complete the questionnaire and return it to the investigators. The questionnaire was designed to collect the following information: (1) socio-demographic characteristics of the women, (2) knowledge and (3) attitudes about the main maternal risk factors in pregnancy, (4) perception of the pregnant women about causing harm to the fetus or newborn baby as a result of their behavior, (5) behavior in the past 30 days of pregnancy and in the 3 months prior to pregnancy and (6) source of information. A ten-point Likert-type scale with responses ranging from 1 (bad) to 10 (very good) was used to assess self-rated health. To test knowledge, the format used was closed-end questions with dichotomous responses (yes or no) for each risk factor. A respondent’s attitude was assessed with an open format “agree”, “uncertain” and “disagree”. Self-rated worry was assessed on a ten-point Likert-type scale with responses ranging from 1 (not worried) to 10 (very worried). To test behaviors, the format used was closed-end questions with dichotomous (yes or no) and open-ended responses. Finally, in the question regarding information sources, respondents could indicate more than one source and could self-rate the utility of the received information about the main risk factors in pregnancy. This was measured on a ten-point Likert-type scale with responses ranging from 1 (not helpful) to 10 (very helpful). A pilot study was carried out with a sample of 20 pregnant women (and their responses were included in the final sample number of the survey). This was to evaluate the comprehensibility of the wording of each question. After the pilot study no changes were made to the questionnaire.

The study protocol and the questionnaire were approved by the Ethical Committee of the Second University of Naples. To obtain written informed consent all participants were given a letter including information about the purpose and the objectives of the study which indicated that participation in the survey was voluntary and that the privacy and confidentiality would be guaranteed. For minors, written informed consent was obtained from the parents on behalf of minors.

### Statistical analysis

Following a descriptive analysis of the sample, multivariate logistic regression models were performed to assess the contribution of the variables to the outcomes of interest. In this study, we identified four primary outcomes of interest: a) knowledge of the main maternal risk factors in pregnancy (alcohol, smoking, passive smoking and obesity) (Model 1), b) belief that alcohol, smoking, passive smoking and obesity can cause harm to the fetus or newborn baby (Model 2), c) the profile of women who smoke during pregnancy (Model 3) and d) information received by women from physicians during ambulatory gynecological examinations about the main risk factors in pregnancy (alcohol, smoking, passive smoking and obesity) (Model 4). In all models, the following independent variables were included: age (continuous, in years), nationality (Italian = 0 other = 1), marital status (unmarried = 0 married = 1), educational level (three categories: middle school or less = 1 high school = 2 baccalaureate/graduate degree = 3), employment status (unemployed/housewife = 0 employed = 1), number of children (none = 0, ≥1 = 1), number of abortions (none = 0, ≥1 = 1), body mass index (four categories: <18.5 = 1, 18.5–24.9 = 2, 25–25.9 = 3, ≥30 = 4), weeks of pregnancy (three categories: 0 to 11 = 1, 12 to 27 = 2, 28 to 41 = 3), self-rated health status (continuous) and need for additional information about the main risk factors in pregnancy (no = 0 yes = 1). The following variables were also included in Models 2, 3 and 4: knowledge of alcohol and smoking as risk factors in pregnancy (no = 0 yes = 1), knowledge of alcohol, smoking and passive smoking as risk factors in pregnancy (no = 0 yes = 1), and knowledge of alcohol, smoking, passive smoking and obesity as risk factors in pregnancy (no = 0 yes = 1). In Models 1, 2 and 3: having received information from the physician about the main maternal risk factors in pregnancy (no = 0 yes = 1) and the usefulness of the information received about the main maternal risk factors in pregnancy (continuous). In Model 3: knowledge of smoking as a risk factor in pregnancy (no = 0 yes = 1), belief about the harm of smoking cigarettes during pregnancy (no = 0 yes = 1) and having received information from the physician about the hazards of smoking in pregnancy (no = 0 yes = 1). In Model 4: smoking during pregnancy (no = 0 yes = 1) and alcohol consumption during pregnancy (no = 0 yes = 1).

The results of the regression analysis are expressed as odds ratios (ORs) and 95% confidence intervals (CIs). A statistically significant level was considered as a two-tailed *p*-value less than 0.05. Statistical analyses were performed using Stata version 10.1 software [32].

## Results

### Participants’ characteristics

In total, 513 questionnaires were distributed and 512 women agreed to participate in the study (response rate 99.8%). The characteristics of the respondents are summarized in Table 1. The average age was 28.5 years, and more than two-thirds of women were married (71.1%). Approximately one-third had completed at least high school education (36.3%), almost all were of Italian nationality (92.6) and one-third of the sample (35.3%) had a body mass index ≥25. Moreover, approximately one quarter of the respondents (26.7%) had undergone at least one abortion, only 21.3% had more than one child and 85.2% of the sample had completed the first trimester of pregnancy.

**Table 1:** Socio-demographic characteristics of the pregnant women.

	Total	
	(*n* = 512)	
	N	%
*Age (years)*	28.5±5.7(17–45)*	
<20	36	7
21–25	134	26.3
26–30	158	31
>30	182	35.7
*Nationality*		
Italian	473	92.6
Other	38	7.4
*Marital status*		
Married	362	71.1
Other	147	28.9
*Educational level*		
Primary school or less	47	9.2
Middle school	220	43.1
High school	185	36.3
Baccalaureate/graduate degree	58	11.4
*Employment status*		
Unemployed/housewife	305	60.9
Employed	196	39.1
*Number of children*		
0	238	46.6
1	164	32.1
>1	109	21.3
*Number of abortions*		
0	362	73.3
1	98	19.8
>1	34	6.9
*Body Mass Index (BMI)*		
<18.5	27	5.4
18.5–24.9	294	59.3
25–25.9	132	26.6
>30	43	8.7
*Weeks of pregnancy*		
0–11	75	14.8
12–27	227	44.7
28–40	206	40.5

Number for each item may not add up to the total number of the study population due to missing values

* Mean±standard deviation (range)

### Participants’ knowledge

When assessing the levels of knowledge, a large percentage of pregnant women knew that alcohol (90.4%), smoking (88.1%), obesity (66.1%) and passive smoke (63.3%) during pregnancy could harm the health of the fetus.

However, only 42% of the sample correctly knew all the main maternal risk factors in pregnancy (alcohol, smoking, passive smoking and obesity). The results of the multiple logistic regression analysis revealed that four variables were significantly associated with knowledge of the main maternal risk factors (Model 1 in Table 2). Respondents who were older (OR = 1.05; CI 95% = 1.02–1.09), those who were very worried with causing harm to the fetus or newborn baby (OR = 1.69; CI 95% = 1.04–2.74) and women who were not of Italian nationality (OR = 0.36; CI 95% = 0.16–0.81) were more likely to know the main maternal risk factors in pregnancy. Moreover, women with a middle school or lower educational level were significantly less likely than women with a baccalaureate degree/graduate degree to know the main maternal risk factors in pregnancy (OR = 0.64; 95% CI = 0.43–0.95).

**Table 2:** Multivariate logistic regression analyses indicating associations between several variables and the different outcomes regarding main risk factors in pregnancy.

**Variable**	**OR**	**SE**	**95% CI**	***p* value**
**Model 1**. Knowledge of the main risk factors in pregnancy				
Log likelihood = -277.59, χ2 = 26.73 (5 df), p<0.0001				
Age	1.05	0.02	1.02–1.09	0.004
Nationality	0.36	0.15	0.16–0.81	0.014
Educational level				
Baccalaureate/graduate degree	1*			
Middle school or less	0.64	0.13	0.43–0.95	0.027
Being very worried about causing harm to the fetus or newborn	1.69	0.42	1.04–2.74	0.036
Physician as source of information	1.32	0.33	0.81–2.17	0.263
**Variable**	**OR**	**SE**	**95% CI**	***p* value**
**Model 2**. Belief that alcohol, smoking, passive smoke and obesity can cause harm to the fetus or newborn				
Log likelihood = -215.78, χ^2^ = 62.61 (8 df), *p*<0.0001				
Knowledge of the main risk factors in pregnancy	4.13	1.02	2.54–6.71	<0.001
Educational level				
Baccalaureate/graduate degree	1*			
Middle school or less	0.45	0.16	0.22–0.91	0.026
High school	0.62	0.22	0.31–1.24	0.180
Need of additional information toward the main risk factors in pregnancy	1.54	0.37	0.96–2.46	0.073
Body Mass Index				
18.5–24.9	1*			
>30	0.4	0.21	0.14–1.13	0.085
Physician as source of information	1.65	0.52	0.88–3.08	0.115
Employment status	0.68	0.17	0.42–1.11	0.129
Self-rated worry	1.46	0.41	0.84–2.54	0.175
**Variable**	**OR**	**SE**	**95% CI**	***p* value**
**Model 3**. Profile of women who smoke during pregnancy				
Log likelihood = -225.62, χ2 = 34.52 (12 df), *p*<0.001				
Having at least one abortion	1.84	0.49	1.09–3.12	0.023
Educational level				
Baccalaureate/graduate degree	1*			
Middle school or less	2.89	1.49	1.05–7.94	0.039
High school	1.67	0.88	0.59–4.72	0.326
Consider very useful information received about risk factors in pregnancy	0.99	0.01	0.99–1.01	0.069
Knowledge of smoking as a risk factor in pregnancy	0.67	0.17	0.41–1.08	0.104
Number of children	1.45	0.38	0.87–2.43	0.154
Age	0.97	0.02	0.92–1.01	0.163
Physician as source of information	0.71	0.19	0.41–1.21	0.206
Marital status	0.73	0.19	0.44–1.21	0.222
Attitudes to smoking in pregnancy	0.89	0.11	0.69–1.14	0.360
Self-rated health status	0.79	0.19	0.49–1.29	0.361
Weeks of pregnancy				
0–11	1*			
28–41	0.8	0.19	0.5–1.3	0.375
**Variable**	**OR**	**SE**	**95% CI**	***p* value**
**Model 4**. Profile of women who have received information by physicians during ambulatory gynecological examinations about main risk factors in pregnancy				
Log likelihood = -297.86, χ^2^ = 42.23 (7 df), *p*<0.0001				
Need of additional information toward the main risk factors in pregnancy	0.41	0.08	0.28–0.62	<0.001
Age	1.07	0.02	1.03–1.11	0.001
Very worried about causing harm to the fetus or child with their risk behaviors	1.6	0.39	0.99–2.57	0.051
Number of children	0.7	0.15	0.46–1.06	0.094
Nationality	0.57	0.23	0.26–1.27	0.170
Marital status	1.34	0.3	0.86–2.09	0.195
Weeks of pregnancy				
0–11	1*			
28–41	0.79	0.16	0.53–1.17	0.247

* Reference category

### Participants’ attitudes

Attitudes regarding the main risk factors in pregnancy indicated that a large proportion of women agreed that consumption of alcohol (91.4%), rubella infection (86.7%), maternal weight (56.2%) and smoking (53.7%) in pregnancy could cause harm to the fetus. Moreover, 90% of women agreed that it is useful to make the TORCH complex test in pregnancy.

Only 25% of women agreed that alcohol, smoking, passive smoking and obesity could result in harm to the fetus or newborn baby. Multivariate logistic regression analysis for this outcome (Model 2 in Table 2) showed that women were more likely to agree if they had knowledge of the main maternal risk factors in pregnancy (OR = 4.13; 95% CI = 2.54–6.71). Furthermore, women with a middle school or lower educational level were significantly less likely to agree that alcohol, smoking, passive smoking and obesity could result in harm to the fetus or newborn baby than those with a baccalaureate degree/graduate degree (OR = 0.45; 95% CI = 0.22–0.91).

Only 21.7% of women were very worried about causing harm to the fetus or the child with their risk behaviors.

### Participants’ behavior

Regarding behavior, 22.3% of women reported smoking during pregnancy with an average consumption of 7.4 cigarettes per day. Approximately one-third of women (28.9%) reported drinking alcohol regularly before pregnancy, but 74.8% of these women reported that they had stopped drinking alcohol when they became pregnant. Only 7.2% of women continued to drink alcohol when pregnant and 27.3% of women who were drinking alcohol during pregnancy had the intention of quitting drinking alcohol (Table 3). Also, 7.6% of women both smoked and drank alcohol during the first trimester of pregnancy. Multiple logistic regression analysis was used to determine the variables associated with the profile of women who smoked during pregnancy (Model 3 in Table 2). The results indicated that this behavior was more likely in women who had undergone at least one abortion (OR = 1.84; 95% CI 1.09–3.12) and in women with a middle school or lower education (OR = 2.89; 95% CI 1.05–7.94) compared with women who had at least a college degree.

**Table 3:** Women’s attitudes and behaviors in pregnancy.

	Total	
	(*n* = 512)	
	N	%
*Regular smoking* ^a^		
No	307	60
Yes	205	40
*Smoking during pregnancy* ^b^		
No	398	77.7
Yes	114	22.3
*Number of cigarettes smoked during pregnancy*	7.4±6.1(1–30)*	
1–9 cigarettes per day	70	63.1
10–19 cigarettes per day	30	27
>20 cigarettes per day	11	9.9
*Regular alcohol drinkers*		
No	364	71.1
Yes	148	28.9
*Alcohol drinker during pregnancy*		
Non drinker	474	92.8
Drinker	37	7.2
*Intention of quitting drinking* ^c^		
No	24	72.7
Yes	9	27.3

Number for each item may not add up to the total number of the study population due to missing values

^a^ Regular smoking was defined as smoking ≥100 cigarettes in a lifetime

^b^ Only for those who smoked regularly

^c^ Only for those who drank alcohol during pregnancy

* Mean±standard deviation (range)

Two-thirds of women (75.4%) indicated that during ambulatory gynecological examinations they had received information from the physician about possible damage to the newborn baby resulting from alcohol intake during pregnancy and almost all women (94%) had been informed of the potential damage caused by cigarette smoking. Moreover, a large proportion of women had been informed by physicians that it was important during pregnancy to control their weight (92.4%), blood pressure (89.7%) and blood glucose level (84.3%). Only 43.7% of pregnant women indicated that during ambulatory gynecological examinations they had received information from the physician about possible damage resulting from all the main risk factors in pregnancy (alcohol, smoking, passive smoking and obesity). The results of multivariate logistic regression analysis for this outcome (Model 4 in Table 2) showed that receiving information from physicians during ambulatory gynecological examinations was more likely among older women (OR = 1.07; 95% CI 1.03–1.11) and those who did not believe that they needed more information about maternal risk factors in pregnancy (OR = 0.41; 95% CI 0.28–0.62).

### Major sources of knowledge

Questions concerning sources of information indicated that 71.6% of women received information about risk factors in pregnancy from physicians. Other sources were television/newspapers (21.3%) and the internet (7.2%). More than one-third of women (40.3%) believed that the information they received about risk factors in pregnancy was very useful and more than half of the respondents believed that they had no need for additional information (61%).

## Discussion

To the best of our knowledge, the present study is one of the few investigations designed to collect detailed data on the knowledge, attitudes and behaviors of pregnant women regarding several maternal risk factors simultaneously.

In this study, the majority of women had adequate knowledge of the main risk factors in pregnancy. Indeed, the results indicate that the majority of women knew that alcohol exposure, smoking, passive smoking and obesity were maternal risk factors during pregnancy. These findings can be compared with those found in other studies. In a study on 499 reproductive-aged women, 95.8% and 98.2%, respectively, knew that alcohol use and smoking were risk factors potentially affecting a pregnancy [33]. In a cross-sectional survey of 305 reproductive-aged women at an urban public hospital. 93% knew that alcohol use during pregnancy could cause birth defects and 86% agreed that smoking should be avoided during pregnancy [34]. In a survey conducted in Pakistan regarding the knowledge of reproductive-aged women about smoking, 77% and 88% of respondents knew that smoking and passive smoking, respectively, could adversely affect the health of the fetus [35]. In a study about tobacco use and exposure to second hand smoke among pregnant women in the Dominican Republic 98% of the sample believed that women who smoked could harm their unborn baby’s health but conversely only 2% believed that exposure to passive smoking could cause illness in children [36]. Finally, in two studies conducted in Australia 94% and 75% of pregnant women knew that obesity would be associated with pregnancy complications [27,28].

In the current study only 42% of the respondents correctly knew all the main maternal risk factors (alcohol exposure, smoking, passive smoking and obesity). Our results suggest that continued efforts are urgently needed to educate women. In the multivariate analysis, age, educational level, nationality and concern about causing harm to the fetus or newborn baby were identified as being significantly associated with knowledge of the main risk factors in pregnancy. Lack of knowledge amongst women with lower education has already been described in another study conducted in Brazil which assessed knowledge of appropriate physical exercise during pregnancy [37].

Attitudes regarding the main risk factors in pregnancy indicated that approximately one-third of women were very worried about causing harm to the fetus or newborn baby with their risk behaviors and only 25% of the sample agreed that alcohol, smoking, passive smoking and obesity could result in harm to the fetus or newborn baby.

Our results provide evidence that pregnant women’s cigarette smoking is a current problem in Italy. Indeed, 22.3% of women reported smoking during pregnancy. This result was higher than that found in three studies conducted previously in Italy where the 6.7%, 8.2%, and 12.2% of the women smoked during pregnancy [5,38,39]. Although the proportion of women who smoked during pregnancy in our study is comparable with two large cross-sectional studies conducted in Canada and in Europe (including Italy), the prevalence of women who smoked during pregnancy was 23% and 26.2% respectively [21,40]. However, the values observed in the current study were significantly different from those found in other studies. In particular, in a study conducted in women who attended antenatal ultrasound scan clinics in the United Kingdom, 57.4% of women reported being current smokers [41] and in Australia 46% of women reported tobacco use during pregnancy [30]. Finally, the latest report on the data of the Certificate of Assistance for Birth of the Emilia Romagna region in Italy reports that the 39.4% of regular smokers continued to smoke during pregnancy [42]. Conversely, in two other studies in Canada and Iceland the observed values were significantly lower for smoking in pregnancy: 5% and 10.5% respectively [22,23].

The results of our study showed that the proportion of women who drank alcohol in pregnancy was only 7.2%. This percentage is significantly lower than that found in other studies, possibly because women in the current study believed that alcohol would adversely affect the health of newborn babies more than smoking [25,29–31].

In the multivariate model to evaluate the profile of women who smoked during pregnancy, only two variables were identified as significantly associated with this behavior: having undergone at least one abortion, having middle school or lower education. This result is consistent with that of two studies conducted in the UK and Iceland which both found a significant association between smoking during pregnancy and low levels of education [22,41]. In addition, a univariate analysis in another study in Canada found that lower levels of education were significantly associated with smoking during pregnancy [21]. Although it is important and more effective to implement measures to promote appropriate behaviours in women before pregnancy, these results suggested that is important to assess the knowledge and behaviors of pregnant women about the main risk factors.

Regarding sources of information, as would be expected, two-thirds of women indicated that they received information from doctors about risk factors in pregnancy; however, the majority of respondents declared that they did not need more information. It is interesting to observe that only 43.7% of the respondents stated that they received information about possible harm associated with all the main risk factors in pregnancy during gynecological examinations. This finding highlights the need for obstetricians to increase the time dedicated to providing information about these very important topics for the health of women and unborn children.

When interpreting the results of this study several limitations should be considered. The first is that this study is a cross-sectional survey. Therefore, the directionality of the association between the independent variables and the different outcomes of interest cannot be determined. The second limitation is that data was collected by self-administered questionnaires and the answers could lead to overestimation or underestimation of perceptions and behaviors. However, this risk was limited by providing written responses and ensuring the anonymity of the respondents. Despite these limitations, this study had a large, properly selected sample and high response rate and therefore the findings may be generalizable.

In conclusion, the results of this study indicate that pregnant women lack knowledge regarding the main maternal risk factors. Pregnant women claim to receive little information during gynecological examinations and, therefore, some continue to smoke and/or drink alcohol during pregnancy. Our results suggest an urgent need to design interventions to improve education levels and appropriate behaviours in relation to the major risk factors in pregnant women.
